# Efficacy of antimalarial treatment in Guinea: *in vivo *study of two artemisinin combination therapies in Dabola and molecular markers of resistance to sulphadoxine-pyrimethamine in N'Zérékoré

**DOI:** 10.1186/1475-2875-6-54

**Published:** 2007-05-03

**Authors:** Maryline Bonnet, Cally Roper, Martine Félix, Léonie Coulibaly, Gabriel Mufuta Kankolongo, Jean Paul Guthmann

**Affiliations:** 1 Epicentre, 8, rue Saint Sabin 75011 Paris, France; 2 Department of Infectious and Tropical Diseases, London School of Hygiene and Tropical Medicine, London, UK; 3 Médecins Sans Frontières, Geneva, Switzerland; 4 National Malaria Control Program, Conakry, Republic of Guinea

## Abstract

**Background:**

In the last five years, countries have been faced with changing their malaria treatment policy to an artemisinin-based combination therapy (ACT), many with no national data on which to base their decision. This is particularly true for a number of West African countries, including Guinea, where these studies were performed. Two studies were conducted in 2004/2005 in programmes supported by Medecins Sans Frontieres, when chloroquine was still national policy, but artesunate (AS)/sulphadoxine-pyrimethamine (SP) had been used in refugee camps for two years.

**Methods:**

In Dabola (central Guinea), 220 children aged 6–59 months with falciparum malaria were randomized to receive either AS/amodiaquine (AQ) or AS/SP. In vivo efficacy was assessed following the 2003 World Health Organization guidelines. In a refugee camp in Laine (south of Guinea), where an in vivo study was not feasible due to the unstable context, a molecular genotyping study in 160 patients assessed the prevalence of mutations in the dihydrofolate reductase (dhfr) (codons 108, 51, 59) and dihydropteroate synthase (dhps) (codons 436, 437, 540) genes of Plasmodium falciparum, which have been associated with resistance to pyrimethamine and sulphadoxine, respectively.

**Results:**

In Dabola, after 28 days of follow-up, Polymerase Chain Reaction (PCR)-adjusted failure rates were 1.0% (95%CI 0–5.3) for AS/AQ and 1.0% (95%CI 0–5.5) for AS/SP. In the refugee camp in Laine, the molecular genotyping study found three dhfr mutations in 85.6% (95%CI 79.2–90.7) patients and quintuple dhfr/dhps mutations in 9.6% (95%CI 5.2–15.9).

**Conclusion:**

Both AS/AQ and AS/SP are highly efficacious in Dabola, whereas there is molecular evidence of established SP resistance in Laine. This supports the choice of the national programme of Guinea to adopt AS/AQ as first line antimalarial treatment. The results highlight the difficulties faced by control programmes, which have gone through the upheaval of implementing ACTs, but cannot predict how long their therapeutic life will be, especially in countries which have chosen drugs also available as monotherapies.

## Background

In the last five years, countries have been faced with changing their malaria treatment policy to an artemisinin-based combination therapy (ACT), many with no national data on which to base their decision. This is particularly true for a number of West African countries; the Republic of Guinea is a good example. Indeed, when the National Malaria Control Programme (NMCP) of Guinea initiated its treatment policy change for treatment of non-severe malaria, replacing chloroquine (CQ) with an ACT, the only data available of antimalarial efficacy were results from two 14 day studies of CQ efficacy conducted in the South of Guinea (N'Zerekore) in 2000 and 2001, which reported failure rates of 24% and 28%, respectively (NMCP, 2001). Nevertheless, high resistance to sulphadoxine-pyrimethamine (SP) and relatively high resistance to AQ were already documented in two neighbouring countries, as high as 52% and 46% for SP and 19% and 30% for AQ in Liberia and Sierra Leone, respectively [1-3].

**Figure 1:**
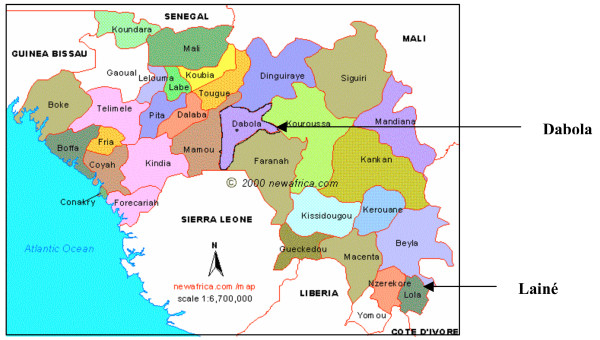
Map of Republic of Guinea and study sites.

Results of studies carried out by Médecins Sans Frontières (MSF) in two different regions of Guinea where the organization is supporting treatment of malaria are reported: an *in vivo *efficacy study which assessed the efficacy of two potential ACT candidates (AS/SP and AS/AQ) to replace the first line CQ treatment in Dabola prefecture (central Guinea) and the assessment of SP resistance from a genotypic analysis in the Lainé Liberian refugee camp (South Guinea) where AS/SP was used since 2002 and where it was not possible due to security reasons to conduct an *in vivo efficacy *study (Figure 1). The Ministry of Health of Guinea approved both studies. Written informed consent was obtained from each included patient, or carer.

## Methods

### *In vivo *study

#### Study site

Dabola prefecture (114,000 inhabitants) has an average altitude of 1,200 m and seasonal perennial malaria, with increased transmission between June and October. *Plasmodium falciparum *is the predominant species. Dabola reported 15,221 clinical malaria cases in 2003, accounting for 30% of the total outpatient consultations (MOH, 2003).

### Inclusion procedure

The efficacy study was based on the last WHO recommendations for the assessment and monitoring of antimalarial drug efficacy for area of high transmission [4]. Children aged 6 to 59 months with measured fever (axillary temperature ≥ 37.5°C) were screened from outpatient lines. Children with a *P. falciparum *mono-infection and asexual parasitaemia between 2,000 and 200,000/μL were eligible for inclusion to the study. Exclusion criteria were (i) signs of severity or severe malaria according to WHO criteria, which included severe anaemia defined by haemoglobin < 5 g/dl [5], (ii) history of allergic reactions to the study drugs, (iii) presence of a concomitant febrile condition with the potential to confound study outcome (e.g. ARI, measles, severe diarrhoea, etc.) and (iv) severe malnutrition [4].

### Treatment and follow-up

Study regimens consisted of either sulphadoxine-pyrimethamine 1.25-mg/Kg stat (Fansidar^®^, Roche, France); or amodiaquine 30 mg/Kg base divided into three daily doses of 10 mg/Kg (Camoquin^®^, Parke-Davis, Senegal); each combined with artesunate at a daily dose of 4 mg/Kg on days 0, 1 and 2 (Arsumax^®^, Guilin Pharmaceutical Works, China). Drugs were crushed and mixed with water and sugar, given in a spoon or a syringe to small children. All doses were directly observed and repeated if vomiting occurred within 30 minutes. Patients were randomly allocated (blocks of 20), without concealment, to receive either AS/SP or AS/AQ.

On day 0, blood samples were taken for haemoglobin measurement, parasitaemia including gametocytaemia and possible genotypic analysis to distinguish recrudescence from re-infection in the event of parasite recurrence. After treatment (days 0, 1, and 2), children were re-assessed clinically and parasitologically on days 3, 7, 14, 21, and 28. Children, who were parasitaemic but asymptomatic during follow-up were asked to return to the clinic every three days. Gametocyte carriage was re-measured at day 28, and a second blood sample for PCR genotyping was collected in case of symptomatic recurrent parasitaemia occurring after day 9 (recurrences on or before day 9 were assumed to be recrudescence, i.e. true failures), or at the end of follow-up in the presence of parasitaemia without symptoms (MSF/Epicentre guidelines). Rescue therapy (quinine hydrochloride 10 mg/Kg/8 hourly for seven days) was administered to treatment failures, orally or parenterally according to the patient's clinical condition.

Children were withdrawn from the study in case of (i) vomiting any study dose twice, (ii) withdrawal of consent, (iii) onset of a serious febrile illness, (iv) intake of any drug with antimalarial properties, (v) missing any treatment dose, (vi) mixed species parasitaemia or (vii) any protocol violation. Patients who missed follow up visits and did not come on the successive day despite tracing were considered lost to follow up. Patients were classified as early treatment failure (ETF), late clinical failure (LCF), late parasitological failure (LPF) or adequate clinical and parasitological response (ACPR) as per WHO definitions [4].

### Laboratory methods

Capillary blood was obtained by fingerprick. Thick and thin films, prepared on the same slide, were stained with 10% Giemsa (pH 7.2) for 15 minutes. Asexual parasitaemia was quantified against 200 to 500 leukocytes, assuming a white blood cell count of 8000/μL [6]. Presence of gametocytes was recorded through this same method. All slides were re-read and any discordance (positivity, species or density for parasitaemia below 2,000 or above 2,000,000/μL) was resolved by a third reader. External quality control on a random sample of 92 slides was carried out by an independent and experienced laboratory technician in Geneva and showed only two negative/positive discordances (2.2%), which did not change the outcome classification. Haemoglobin was measured using the Lovibond technique (Assistant Co., Sondheim Rhon, Germany). Anaemia was defined as haemoglobin (Hb) < 11 g/dl [7].

Blood samples for PCR analysis were collected on Isocode^® ^kits (Schleicher & Schuell, Ecquevilly, France). Kits were air dried and stored in cool and dark boxes with desiccants. Genotypic analysis was performed at the Epicentre laboratory at Mbarara University (Uganda) according to a published method considering the three *P. falciparum *gene loci merozoite surface protein-1 (*msp-1*), merozoite surface protein-2 (*msp-2*), and glutamate rich protein (*GLURP*) [8]. Cases in which pre- and post-treatment genotypes were identical were considered as recrudescence, i.e. failures; cases in which pre- and post-treatment genotypes were different were considered as re-infections; mixed genotypes were classified as failures.

### Sample size

As no reliable estimates of antimalarial efficacy were available, all our calculations were based on an estimated 50% failure rate and a desired precision of 10%. Allowing for a type-1 error of 0.05 and considering a 15% drop out rate, 110 patients per study group were needed.

### Data entry and analysis

Records were entered in Epidata 3.0 (The Epidata association, Odense Denmark) and analysed on SPSS^® ^11.0 for Windows (SPSS Inc. Chicago, Illinois) and EpiInfo 6.04b (CDC, Atlanta; 1996). Data entry errors were checked individually on each record. Appropriate statistical tests (Chi2, ANOVA, Kruskal-Wallis were used to compare the two groups. The main outcome was day-28 failure rate, defined as the number of true failures (recurrences on or before day 9 + PCR-confirmed recrudescence occurring after day 9) over the total number of patients analysed. Losses to follow-up and withdrawn patients, as well as PCR-confirmed new infections and indeterminate PCR results were excluded from the analysis according to the last WHO guideline for assessment and monitoring of antimalarial efficacy [4].

### Genotypic analysis

Lainé refugee camp (22,000 Liberian refugees and average altitude 470 m) borders Ivory Cost and Liberia. Malaria transmission is perennial. A total of 10,541 malaria confirmed cases were recorded in 2004 (Figure 1).

Patients from the outpatient clinic of the Lainé refugee camp with measured fever (axillary temperature ≥ 37.5°C) were screened for falciparum malaria using the Paracheck-Pf^® ^rapid diagnostic test. After microscopic confirmation, samples from 181 consecutive patients were collected on Isocode^® ^kits and transported to the London School of Hygiene and Tropical Medicine (LSHTM, London, United Kingdom). After DNA extraction, the PCR-Sequence Specific Oligonucleotide Probing (PCR-SSOP) method was used for molecular genotyping of point mutations in the dihydrofolate reductase (*dhfr*) and dyhydropteroate synthetase (*dhps*) genes [9,10]. This method involves the PCR amplification of coding regions of *dhfr *and *dhps *genes, which are fixed onto membranes and probed with SSOP designed to detect each of the single base-pair changes which code for substitutions at codons 51 (N to I), 59 (C to R), and 108 (S to N, or T) of *dhfr *and codons 436 (S to A, or F), 437 (A to G), and 540 (K to E) of *dhps*. Since point mutations occur in a number of different configurations, each conferring differing degrees of resistance, data were to summarized in two ways. Firstly, by showing the allelic haplotypes (combination of mutations which are in the same gene), which were found among the parasites in Lainé. These can be determined easily in infections where there is a single genotype because the blood stage form of the parasite is haploid but are difficult or impossible to resolve in mixed infections [9,10]. The frequency of allelic haplotypes in the parasite population was calculated after excluding mixed infections. The second way in which were summariszd the data, was to calculate the proportion of all infections (mixed and non-mixed), which were found to contain all three *dhfr *mutations plus the *dhps *437 and 540 mutations. This particular genotype has been shown to be associated with SP treatment failure in a number of East African countries: Kenya, Malawi and Uganda but has only been reported rarely in West Africa [11-13].

## Results

### Efficacy study

**Figure 2:**
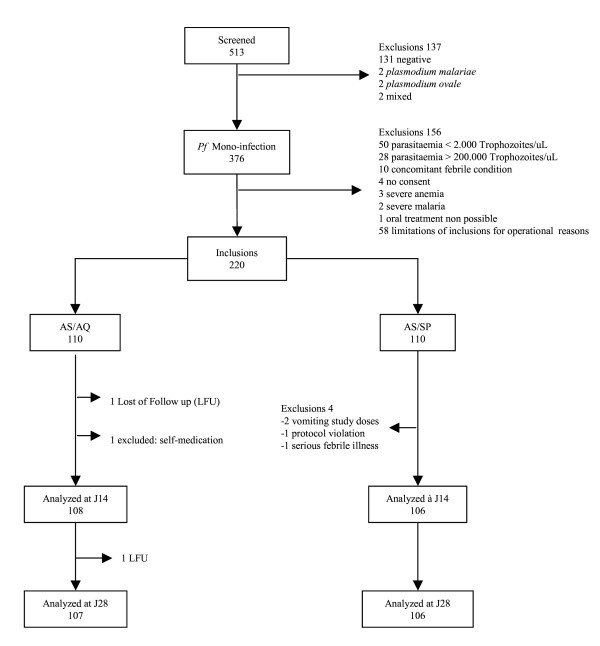
Details on study inclusions and follow-up.

Between June and September 2004, 513 children were screened, of whom 376 (73.9%) had a *P. falciparum *mono-infection and 220 (42.2%) were included (Figure 2). Of the 220 patients included, 2 (0.9%) were lost to follow-up (AS/AQ group) and 5 (2.3%) withdrawn (1 in the AS/AQ group, 4 in the AS/SP group). Baseline characteristics were similar across treatment groups (Table 1). After 28 days of follow-up, there were 6/107 (5.6%) recurrent parasitaemia in the AS/AQ group and 9/106 (8.5%) in the AS/SP group (p = 0.41). Among these 15 recurrences, one was an ETF (true failure, AS/SP) and 14 were late failures that occurred after day 9. Out of these 14 samples that underwent PCR genotyping, 13 had a result available: 12 were re-infections excluded from the analysis (five AS/AQ, seven AS/SP) and one was a recrudescence (true failure, AS/AQ). The 28-day PCR-adjusted failure rate was, therefore, 1/102 (1.0%, 95%CI 0–5.3) in the AS/AQ group and 1/99 (1.0%, 95%CI 0–5.5) in the AS/SP group (Table 2). After the end of treatment (day 3), 95.4% (105/110, AS/AQ) and 96.3% (103/107, AS/SP) of the patients had cleared their parasitaemia (p = 0.15). Compared to baseline, gametocyte carriage was significantly decreased after 28 days of follow-up in both groups (from 11.8% (13/110) to 3.8% (4/106) in the AS/AQ group; from 10.0% (11/110) to 2.0% (2/101) in the AS/SP group; p = 0.03 and p = 0.01 for each comparison, respectively).

**Table 1:** Baseline characteristics of included patients in the *in vivo *study, Dabola

**Characteristics**		**AS/AQ (n = 110)**	**AS/SP (n = 110)**	**p**
**Gender ratio**	M/F	54/56 0.96	51/59 0.86	0.16
**Age (months)**	Mean (SD)	29.5 (14.9)	28.7 (12.9)	0.68
**Middle-upper arm circumference (mm)**	Mean (SD)	149.2 (12.5)	147.6 (10.8)	0.29
**Axillary temperature (°C)**	Median (IQR)	39.0 (37.8–39.8)	39.1 (38.1–40.0)	0.46
**Haemoglobin (g/dL)**	Mean (SD)	8.7 (2.0)	8.7 (1.9)	0.92
**Asexual parasitaemia (/μL)**	Geometric mean (IQR)	40,492 20,121–97,615	34,716 15,658–41,226	0.45
**Gametocyte carriage**	n (%) 95% CI	13 (11.8) 6.4 – 19.3	11 (10.0) 5.1 – 17.8	0.66

SD: Standard deviation IQR: Inter-Quartile Range

**Table 2:** Therapeutic response at day 28, after PCR adjustment, *in vivo *study, Dabola

**Results**	**AS/AQ (n = 102)**			**AS/SP (n = 99)**		
	N	%	95%CI	N	%	95%CI
**Early Treatment failure**	0	0	0–3.5	1	1	0–5.5
**Late Treatment Failure**	1	1.0	0–3.5	0	0	0–3.7
**Total failure**	1	1.0	0–5.3	1	1.0	0–5.5
**ACPR**	101	99.0	94.7–99.8	98	99.0	94.5–99.8

Outcome classification according to WHO guidelines [4]

Late Treatment Failure = Late Clinical Failure + Late Parasitological Failure

ACPR: Adequate Clinical and Parasitological Response

Outcome classification is WHO (2003)

### Genotypic analysis

Among the 181 samples sent for analysis, there was no amplification of the coding regions of *dhfr *in 21 cases (11.6%) or of *dhps *in 45 cases (24.9%). One analysis was incomplete for the coding regions of *dhps*. Results were reported for 160 samples for *dhfr *mutations at all of three codons 108, 51 and 59, 135 samples for *dhps *mutations at codons 436, 437 and 540 and for 135 samples for combined *dhfr-dhps *mutations (Tables 3 and 4). Table 3 shows the frequency of allelic haplotypes among the parasite population and this is based upon a subset of infections which were either single or majority genotype infections *dhps *n = 114, *dhfr *n = 148 and *dhfr-dhps *combined n = 110. The proportion of infections which contained all three *dhfr *mutations whether mixed or not, was 85.6% (137/160, 95%CI 79.2–90.7); the proportion containing double mutant *dhps *(437, 540) was 10.4% (14/135, 95%CI 5.8–16.8); and the proportion containing all five was 9.6% (13/135, 95%CI 5.2–15.9) (Table 4).

**Table 3:** Frequency of *dhfr *and *dhps *mutant alleles, genotypic study, Lainé refugee camp

**Genotype Frequencies**	n	%	95% CI
***dhps *(N = 114)**			
wild type,	8	7.0	3.1–13.6
single mutant 436	31	27.2	19.3–36.3
single mutant 437	38	33.3	24.8–42.8
double mutant 436–437	29	25.4	17.7–34.4
double mutant 437–540	8	7.0	3.1–13.6
			
***dhfr *(N = 148)**			
wildtype	20	13.5	8.4–20.1
double mutant 59–108	2	1.4	0.2–4.8
triple mutant 51–59–108	126	85.1	78.4–90.4
			
***dhps *and *dhfr *combined (N = 110)**			
quintuple mutant: *dhfr *51–59–108 &*dhps *437–540	8	7.3	3.2–13.8
quintuple mutant: *dhfr *51–59–108 &* dhps *436–437	27	24.5	16.8–33.7
*dhfr *51–59–108 &* dhps *single mutant	54	49.1	39.4–58.8
Three or less mutations	21	19.1	12.2–27.7

Dhfr: dihydrofolate reductase

Dhps: dihydropterase synthetase

**Table 4:** Frequency of individuals with molecular makers associated to SP resistance, Lainé refugee camp

Prevalence	No of individuals (Single+majority+mixed aplotypes)	%	95% CI
***dhps *(N = 135)**			
Double mutant 437&540	14 (6+2+6)	10.4	5.8–16.8
Double mutant 436&437	36 (22+7+7)	26.7	19.4–35.0
			
***dhfr *(N = 160)**			
triple mutant 51–59–108	137 (121+5+11)	85.6	79.2–90.7
			
***dhfr *and *dhps *(N = 135)**			
triple mutant 51–59–108 with double mutant 437&540	13 (6+2+5)	9.6	5.2–15.9

Single aplotype: only one single nucleotide polymorphism (SNP) was present

Majority aplotype: volume minority SNP value was more than half of the volume of the majority SNP

Mixed aplotype: volume minority SNP value was less than half of the volume of the majority SNP [9]

## Discussion

The *in vivo *study in Dabola provides important data on the efficacy of SP and AQ when used in combination with an artemisinin derivative in a country where very limited information on antimalarial drug efficacy was available. The NMCP of Guinea recently decided to adopt AS/AQ as first line antimalarial treatment for uncomplicated malaria to replace CQ (Roll Back Malaria 2006). In Dabola prefecture, based on the very good efficacy described here (above 95%) both AS/SP or AS/AQ are very good alternatives to CQ. Although important, these results provide information applicable only to the local area where the studies were conducted, and caution should be taken before extrapolating the results to the whole country. Site-specific patterns of health service utilization or malaria transmission intensity may result geographic differences in the efficacy of a drug [14].

The results of the genotypic study in Lainé are also important because they document the level of resistance to SP in a setting where day-28 *in vivo *efficacy study was not feasible due to the unstable context related to security factors and where the combination AS/SP was already used since two years as first line treatment. Although, the genotypic findings cannot be correlated with any in vivo data from the area and that their predictive value for estimating how long the efficacy of SP used in combination with AS can be sustained is unknown, it was important to be able to document the presence and frequency of molecular markers of SP resistance in this setting. The frequency of the quintuple mutant *dhfr*-*dhps *genotype was quite high 7.3% (and its prevalence among patients was 9.6%). In Africa, the *dhfr *triple mutant genotype (108, 51 and 59), which is a resistance marker of pyrimethamine has been shown to be strongly associated with resistance to SP, and this association is strengthened when the triple mutation is associated to the double *dhps *mutant (437, 540), the quintuple mutation being the most predictive marker of treatment failure [12]. These results in Lainé are consistent with the high failure rate of SP from *in vivo *studies in neighbouring Liberia and Sierra Leone [1,2]. The results of Lainé are likely to be specific to that area, since the study population was composed of Liberian refugees living in different conditions from the native population in the surrounding area and with access to AS/SP, whereas in other areas of the country patients were treated with CQ. Therefore, these results were of limited relevance in guiding the national programme in their choice of ACT.

In countries with no or very limited efficacy data of monotherapies (SP and AQ) or ACTs, such as in Guinea, choosing a standard ACT suitable for the whole country is difficult. The choice is usually based on a pragmatic decision, taking into consideration the potential risk of low efficacy based on data from neighbouring countries, such as Liberia and Sierra Leone in the Guinea case, availability and affordability of the ACT, which is still a limitation for artemether-lumefantrine and other advantages and disadvantages of the remaining ACT candidates such as dosing regimen and tolerance. The advantages of AS/SP and AS/AQ have been well described [15,16]. Their price, when available in blister, is similar, about 1.5 US$ each. AS/SP has the advantage that SP is administrated as a single dose and can be delivered to pregnant women during 2^nd ^and 3^rd ^trimesters. Therefore, considering the high levels of resistance to SP in neighbouring countries [1-3], the rather short useful therapeutic lifespan of SP-containing combinations [14] and the fixed combination of AQ/AS just registered in Morocco, AS/AQ seems to have been a good choice. These advantages may explain why other African countries such as Burundi, Cameroon, Côte d'Ivoire, DRC, Equatorial Guinea, Gabon, Ghana, Liberia, Madagascar, Senegal, Sierra Leone and Sudan have already adopted AS/AQ as first-line therapy (Roll Back Malaria 2006).

Concurrent in vivo and molecular studies of AS-SP are needed to see if there is a correlation between molecular findings and *in vivo *response. Genotypic studies are much simpler to perform than *in vivo *studies, particularly in countries in unstable situations. The predictive value of molecular results for estimating how long the useful therapeutic life of SP used in combination with artesunate should be further investigated.

## Authors' contributions

- MB designed the study, analysed and interpreted the data, wrote this paper

- CR supervised the genotypic analysis, help to the interpretation of the molecular findings and revised the paper

- MF implemented and coordinated the study laboratory procedures in Dabola and revised the paper

- LC helped to design the study, revised the paper

- GMG coordinated the study in the field, supervised all steps of the study including data entry and revised the paper

- JPG supervised the analysis, the interpretation of data and revise the paper.

All authors read and approved the final manuscript.
